# ﻿Phylogenetic, ecological and morphological characteristics reveal two new spider-associated genera in Clavicipitaceae

**DOI:** 10.3897/mycokeys.91.86812

**Published:** 2022-07-07

**Authors:** Wan-Hao Chen, Jian-Dong Liang, Xiu-Xiu Ren, Jie-Hong Zhao, Yan-Feng Han, Zong-Qi Liang

**Affiliations:** 1 Center for Mycomedicine Research, Basic Medical School, Guizhou University of Traditional Chinese Medicine, Guiyang 550025, Guizhou, China Guizhou University of Traditional Chinese Medicine Guiyang China; 2 Institute of Fungus Resources, Department of Ecology, College of Life Sciences, Guizhou University, Guiyang 550025, Guizhou, China Guizhou University Guiyang China

**Keywords:** Clavicipitaceae, convergent evolution, morphology, nutritional preference, phylogeny

## Abstract

Clavicipitaceous fungi are pathogenic to scale insects, white flies and other insect orders. However, a few species are spider-associated. Two new genera from China, *Neoaraneomyces* and *Pseudometarhizium*, are described based on phylogenetic, ecological and morphological characteristics. Two spider-associated species, *Neoaraneomycesaraneicola*, *Pseudometarhiziumaraneogenum*, and an insect-associated species *Pseudometarhiziumlepidopterorum* are included. The morphological characteristics of paecilomyces-like conidiogenous structures, present in many insect/spiders associated species make species-level identifications difficult. A phylogenetic analysis of the combined dataset (ITS, LSU, RPB2 and TEF), placed the two new genera in Clavicipitaceae. The new spider-associated species may be the result of convergent evolution to adapt to the ecological environment and may have undergone host jumping or altered their nutritional preferences.

## ﻿Introduction

Araneogenous or araneopathogenic fungi are spider-pathogenic fungi (Evans and Samson 1987) and found in diverse habitats, such as different kinds of monocot, dicot or coniferous plants including trees, grasses, bamboo, mosses, ferns, and lichens (Shrestha et al. 2019). The known araneogenous fungal genera include *Cordyceps* Fr., and the related anamorphic genera *Akanthomyces* Lebert, *Beauveria* Vuill., *Clathroconium* Samson & H.C. Evans, *Clonostachys* Corda, *Gibellula* Cavara, *Hevansia* Luangsa-ard, Hywel-Jones & Spatafora, *Hirsutella* Pat., *Hymenostilbe* Petch, *Nomuraea* Maubl. and *Purpureocillium* Luangsaard, Hywel-Jones, Houbraken & Samson (Chen et al. 2018). Shrestha et al. (2019) noted that araneogenous fungi are restricted to Cordycipitaceae and Ophiocordycipitaceae, with one exception in Bionectriaceae; there is no report to date of araneogenous fungi in the family Clavicipitaceae within Hypocreales.

Members of Clavicipitaceae are distributed worldwide and found in almost all terrestrial ecosystems. Currently, Clavicipitaceae contains 49 genera and over 500 species (Hyde et al. 2020; Mongkolsamrit et al. 2020; Gao et al. 2021). Among these genera, *Claviceps* Tul. and *Balansia* Speg. are pathogenic only to plants (Diehl 1950). *Pochonia* Bat. & O.M. Fonseca and *Rotiferophthora* G.L. Barron are pathogenic to a wide variety of invertebrates. Seven sexually reproductive genera, *Aschersonia* Mont. (*Hypocrella*), *Conoideocrella* D. Johnson, G.H. Sung, Hywel-Jones & Spatafora, *Orbiocrella* D. Johnson, G.H. Sung, Hywel-Jones & Spatafora, *Regiocrella* P. Chaverri & K.T. Hodge, *Samuelsia* P. Chaverri & K.T. Hodge and *Moelleriella* Bres. are pathogenic to scale insects and white flies (Hemiptera), while Metarhizium (Metarcordyceps) has a broad host association (Luangsa-ard et al. 2017).

During a survey of entomopathogenic fungi and their allies in southwestern China, infected insect and spider specimens were obtained, and some fungal strains were isolated and purified. The goal of this research is to identify those new strains by multigene phylogeny, morphological and ecological characteristics.

## ﻿Materials and methods

### ﻿Specimen collection and identification

Four infected insect and spider specimens (DY10171, DY10174, DY10180 and SD0536) were collected from Duyun City (26°21'24.71"N, 107°22'48.22"E) and Sandu County (25°57'22.21"N, 107°57'54.69"E), Guizhou Province, on 1 October and 1 May, 2019. Isolation of strains was conducted as described by Chen et al. (2019). Fungal colonies emerging from specimens were isolated and cultured at 25 °C for 14 days under 12 h light/12 h dark conditions following protocols described by Zou et al. (2010). The specimens and the isolated living strains were deposited in the Institute of Fungus Resources, Guizhou University (formally Herbarium of Guizhou Agricultural College; code, GZAC), Guiyang City, Guizhou, China.

Macroscopic and microscopic morphological characteristics of the fungi were examined, especially for the arrangement, shape and measurement of phialides and conidia, and also the growth rates were determined from cultures grown on potato dextrose agar (PDA) cultures incubated at 25 °C for 14 days. Hyphae and conidiogenous structures were mounted in lactophenol cotton blue or 20% lactic acid solution and observed with an optical microscope (OM, DM4 B, Leica, Germany).

### ﻿DNA extraction, polymerase chain reaction amplification and nucleotide sequencing

DNA extraction was carried out by Fungal genomic DNA Extraction Kit (DP2033, BioTeke Corporation) in accordance with Liang et al. (2011). The extracted DNA was stored at −20 °C. The amplification of internal transcribed spacer (ITS) region, large subunit ribosomal RNA (LSU) gene, RNA polymerase II largest subunit 2 (RPB2) and translation elongation factor 1 alpha (TEF) by PCR was as described by White et al. (1990), Rakotonirainy et al. (1994), Castlebury et al. (2004) and van den Brink et al. (2012), respectively. Primer sequence information is shown in Suppl. material 1. PCR products were purified and sequenced at Sangon Biotech (Shanghai) Co. The resulting sequences were submitted to GenBank (Table 1).

**Table 1.** List of strains and GenBank accession numbers of sequences used in this study.

Species	Strain No.	GenBank Accession No.			
		ITS	LSU	RPB2	TEF
*Aciculosporiumoplismeni*	MAFF 246966	LC571760	LC571760	LC572054	LC572040
*A.take*	MAFF 241224	LC571753	LC571753	LC572048	LC572034
*A.take*	TNS-F-60465	LC571755	LC571756	LC572049	LC572035
*Akanthomycesaculeatus*	HUA 772	KC519371	-	-	KC519366
*Aschersoniabadia*	BCC 8105	-	DQ518752	DQ522411	DQ522317
*A.placenta*	BCC 7869	-	EF469074	EF469104	EF469056
*Atkinsonellahypoxylon*	B4728	-	-	KP689514	KP689546
*Balansiaepichloe*	A.E.G. 96-15a	-	-	EF468908	EF468743
*B.henningsiana*	GAM 16112	-	AY545727	DQ522413	AY489610
*B.pilulaeformis*	A.E.G. 94-2	-	AF543788	DQ522414	DQ522319
*Bionectriaochroleuca*	AFTOL-ID 187	-	DQ862027	DQ862013	DQ862029
*B.vesiculosa*	HMAS 183151	HM050304	HM050302	-	-
*Calcarisporiumarbuscula*	CBS 221.73	AY271809	-	-	-
*C.arbuscula*	CBS 900.68	KT945003	KX442598	KX442597	KX442596
*C.cordycipiticola*	CGMCC 3.17905	KT944999	KX442599	KX442594	KX442593
*C.cordycipiticola*	CGMCC 3.17904	KT945001	KX442604	KX442607	KX442605
*C.xylariicola*	HMAS 276836	KX442603	KX442601	KX442606	KX442595
*Calonectriailicicola*	CBS 190.50	GQ280605	GQ280727	KM232307	AY725726
*Cephalosporiumcurtipes*	CBS 154.61	AJ292404	AF339548	EF468947	EF468802
*Clavicepsfusiformis*	ATCC 26019	JN049817	U17402	-	DQ522320
*C.purpurea*	GAM 12885	-	AF543789	DQ522417	AF543778
*C.purpurea*	S.A. cp11	-	EF469075	EF469105	EF469058
*Clonostachysrosea*	GJS90-227	-	AY489716	-	AY489611
*Cocoonihabitussinensis*	HMAS254523	KY924870	KY924869	-	-
*C.sinensis*	HMAS254524	MF687395	MF687396	-	-
*Collarinaaurantiaca*	FMR 11134	KJ807178	KJ807181	-	-
*C.aurantiaca*	FMR 11784	KJ807177	KJ807180	-	-
*Conoideocrellaluteorostrata*	NHJ 11343	-	EF468850	-	EF468801
*C.luteorostrata*	NHJ 12516	-	EF468849	-	EF468800
*C.tenuis*	NHJ 6293	-	EU369044	EU369087	EU369029
*Corallocytostromaornithocopreoides*	WAC 8705	-	-	LT216620	LT216546
*Cordycepsbrongniartii*	BCC16585	JN049867	JF415967	JF415991	JF416009
*C.militaris*	OSC93623	JN049825	AY184966	-	DQ522332
*Dactylonectriaalcacerensis*	CBS 129087	JF735333	KM231629	-	JF735819
Dussiellatuberiformis*		-	-	JQ257020	JQ257027
*Elaphocordycepsophioglossoides*	NBRC 106332	JN943322	JN941409	-	-
*E.paradoxa*	NBRC 106958	JN943324	JN941411	-	-
*Ephelisjaponica*	CBS 236.64	MH858427	-	-	-
*E.japonica*	Eph.oryzae	AB038564	-	-	-
*E.tripsaci*	CBS 857.72	NR_153997	NG_059240	-	-
*Epichloeelymi*	C. Schardl 760	-	AY986924	-	AY986951
*E.typhina*	ATCC 56429	JN049832	U17396	DQ522440	AF543777
*Flammocladiellaaceris*	CPC 24422	KR611883	KR611901	-	-
*Fusariumcircinatum*	CBS 405.97	U61677	-	JX171623	KM231943
*F.sublunatum*	CBS 189.34	HQ897830	KM231680	-	-
*Gelasinosporatetrasperma*	AFTOL-ID 1287	-	DQ470980	DQ470932	DQ471103
*Haptocilliumsinense*	CBS 567.95	AJ292417	AF339545	-	-
*Helicocollumkrabiensis*	BCC 71374	-	KT222327	-	KT222342
*H.surathaniensis*	BCC 34463	-	KT222328	-	KT222336
*H.surathaniensis*	BCC 34464	-	KT222329	-	KT222337
*Heteroepichloebambusae*	Ba-01	AB065426	-	-	-
*H.bambusae*	Bo-01	AB065428	-	-	-
*H.sasae*	E.sasae-H	AB065432	-	-	-
*H.sasae*	E.sasae-N	AB065431	-	-	-
*Hydropisphaeraerubescens*	ATCC 36093	-	AF193230	AY545731	DQ518174
*H.lutea*	ATCC 208838	-	AF543791	DQ522446	AF543781
*H.peziza*	GJS92-101	-	AY489730	-	AY489625
*H.rufa*	DAOM JBT1003	JN942883	JN938865	-	-
*Hypocreaamericana*	AFTO -ID 52	DQ491488	AY544649	-	DQ471043
*Hypocrelladiscoidea*	BCC 8237	JN049840	DQ384937	DQ452461	DQ384977
*Hypomycespolyporinus*	ATCC 76479	-	AF543793	-	AF543784
*H.aurantius*	GJS74-69	FJ442642	HM466684	FJ442744	FJ467643
*Keithomyces* sp.	CBS 126563	-	MT078856	-	MT078921
*K.carneus*	CBS 239.32	NR_131993	NG_057769	EF468938	EF468789
*Lecanicilliumattenuatum*	CBS 402.78	AJ292434	AF339565	EF468935	EF468782
*L.lecanii*	CBS 101247	JN049836	KM283794	KM283859	DQ522359
*L.psalliotae*	CBS 367.86	-	KM283800	-	KM283823
*Marquandomycesmarquandii*	CBS 182.27	NR_131994	EF468845	EF468942	EF468793
*Marquandomyces* sp.	CBS 127132	-	MT078857	MT078922	-
*Metapochoniabulbillosa*	CBS 145.70	-	AF339542	EF468943	EF468796
*M.gonioides*	CBS 891.72	AJ292409	AF339550	DQ522458	DQ522354
*M.rubescens*	CBS 464.88	-	AF339566	EF468944	EF468797
*M.sulchlasporia*	CBS 251.83	NR_154139	MH873311	-	KJ398790
*Metarhiziopsismicrospora*	CEHS133a	EF464589	EF464571	-	-
*M.microspora*	INEHS133a	EF464583	EF464572	-	-
*Metarhiziumanisopliae*	ARSEF 7487	-	-	DQ468370	DQ463996
*M.anisopliae*	CBS 130.71	MT078884	MT078853	MT078918	MT078845
*M.flavoviride*	CBS 125.65	MT078885	MT078854	MT078919	MT078846
*M.flavoviride*	CBS 700.74	-	MT078855	MT078920	MT078847
*M.flavoviride*	CBS 218.56	-	-	-	KJ398787
*Moelleriellaphyllogena*	CUP 067785	-	EU392610	-	EU392674
*M.phyllogena*	CUP 067793	-	EU392608	-	EU392672
*M.schizostachyi*	BCC 14123	-	DQ518771	DQ522447	DQ522346
*M.umbospora*	CUP 067817	-	EU392628	-	EU392688
*Mycophilomycespericoniae*	CPC 27558	NR_154209	NG_059746	-	-
*Myriogenosporaatramentosa*	A.E.G 96-32	-	AY489733	DQ522455	AY489628
*Myrotheciomycescorymbiae*	CPC 33206	NR_160351	NG_064542	-	-
*Myrotheciuminundatum*	IMI158855	-	AY489731	-	AY489626
*M.roridum*	ATCC 16297	-	AY489708	-	AY489603
*M.verrucaria*	ATCC 9095	-	AY489713	-	AY489608
*Nectriacinnabarina*	CBS 125165	HM484548	HM484562	KM232402	HM484527
*N.nigrescens*	CBS 125148	HM484707	HM484720	KM232403	HM484672
*Nectriopsisviolacea*	CBS 424.64	-	AY489719	-	-
*Neoaraneomycesaraneicola*	DY101711	MW730520	MW730609	MW753026	MW753033
*N.araneicola*	DY101712	MW730522	MW730610	MW753027	MW753034
*Neobaryaparasitica*	Marson s/n	KP899626	KP899626	-	-
*Neonectriacandida*	CBS 151.29	JF735313	AY677333	-	JF735791
*N.faginata*	CBS 217.67	HQ840385	HQ840382	DQ789797	JF268746
*N.neomacrospora*	CBS 118984	HQ840388	HQ840379	DQ789810	JF268754
*N.ramulariae*	CBS 182.36	HM054157	HM042435	DQ789793	HM054092
*Neurosporacrassa*	ICMP 6360	AY681193	AY681158	-	-
*Niessliaexilis*	CBS 560.74	-	AY489720	-	AY489614
*Nigeliaaurantiaca*	BCC13019	-	GU979948	GU979971	GU979957
*N.martiale*	EFCC 6863	-	JF415974	-	JF416016
*Ophiocordycepsheteropoda*	EFCC 10125	JN049852	EF468812	EF468914	EF468752
*O.sinensis*	EFCC 7287	JN049854	EF468827	EF468924	EF468767
*O.stylophor*	OSC 111000	JN049828	DQ518766	DQ522433	DQ522337
*Orbiocrellapetchii*	NHJ 6209	-	EU369039	EU369081	EU369023
*O.petchii*	NHJ 6240	-	EU369038	EU369082	EU369022
*Papiliomycesliangshanensis*	EFCC 1452	-	EF468815	-	EF468756
*P.liangshanensis*	EFCC 1523	-	EF468814	EF468918	EF468755
*P.shibinensis*	GZUH SB13050311	NR154178	-	-	KR153589
*Parametarhiziumchangbaiense*	CGMCC 19143	MN589741	MN589994	MT921829	MN908589
*P.hingganense*	CGMCC 19144	MN055703	MN061635	MT939494	MN065770
*Parepichloecinerea*	Ne-01	AB065425	-	-	-
*Peethambaraspirostriata*	CBS110115	-	AY489724	EF692516	AY489619
*Periglandulaipomoeae*	IasaF13	-	-	KP689517	KP689568
*Pochoniaboninensis*	JCM 18597	AB709858	AB709831	AB758693	AB758463
*P.globispora*	CBS 203.86	DQ516079	-	-	-
*Pseudometarhiziumaraneogenum*	DY101741	MW730532	MW730618	MW753030	MW753037
*P.araneogenum*	DY101742	MW730534	MW730619	MW753031	MW753038
*P.araneogenum*	DY101801	MW730536	MW730623	MW753032	MW753039
*P.araneogenum*	DY101802	MW730545	MW730625	-	MW753040
*P.lepidopterorum*	SD05361	MW730543	MW730624	-	MW753041
*P.lepidopterorum*	SD05362	MW730611	MW730629	-	MW753042
*Purpureocilliumlavendulum*	FMR 10376	-	FR775489	-	FR775516
*P.lilacinus*	CBS 284.36	-	-	EF468941	EF468792
*Purpureomycesmaesotensis*	BCC 88441	MN781916	MN781877	MN781824	MN781734
*P.maesotensis*	BCC 85349	MN781928	MN781872	-	MN781729
*P.maesotensis*	BCC 89300	MN781917	MN781876	-	MN781733
*Regiocrellacamerunensis*	ARSEF 7682	-	DQ118735	-	DQ118743
*Romanoaterricola*	WCM_17	KP794435	-	-	-
*R.terricola*	WCM_18	KP794436	-	-	-
*Rosasphaeriamoravica*	LMM	JF440985	-	JF440986	JF440987
*Rotiferophthoraangustispora*	CBS 101437	-	AF339535	DQ522460	AF543776
*Roumegueriellarufula*	CBS 346.85	-	DQ518776	DQ522461	DQ522355
*R.rufula*	GJS 91-164	-	EF469082	EF469116	EF469070
*Samuelsiachalalensis*	CUP 067856	-	EU392637	-	EU392691
*S.mundiveteris*	BCC 40021	-	GU552152	-	GU552145
*S.rufobrunnea*	CUP 067858	-	AY986918	-	AY986944
*Sarocladiumbacillisporum*	CBS 425.67	NR_145039	MH870718	-	-
*S.dejongiae*	CBS 144929	NR_161153	NG_067854	-	-
*S.implicatum*	CBS 959.72	HG965023	MH878470	-	-
*S.subulatum*	CBS 217.35	MH855652	NG_070566	-	-
*S.terricola*	CBS 243.59	MH857853	MH869389	-	-
*Shimizuomycesparadoxus*	EFCC 6279	JN049847	EF469084	EF469117	EF469071
*S.paradoxus*	EFCC 6564	-	EF469083	EF469118	EF469072
*Simplicilliumlamellicola*	CBS 116.25	AJ292393	MH866307	DQ522462	DQ522356
*S.lanosoniveum*	CBS 101267	AJ292395	-	DQ522463	DQ522357
*S.lanosoniveum*	CBS 704.86	AJ292396	AF339553	DQ522464	DQ522358
*Sordariafimicola*	AFTOL-ID 216	DQ518178	-	-	DQ518175
*Stachybotryseucylindrospora*	ATCC 18851	JN942887	JN938869	-	-
*Sphaerostilbellaaureonitens*	GJS74-87	FJ442633	HM466683	FJ442763	-
*S.berkeleyana*	GJS82-274	-	U00756	-	AF543783
*S.chlorohalonata*	DAOM 235557	JN942888	JN938870	-	-
*Stachybotrysmicrospora*	CBS 186.79	-	-	DQ676580	DQ676604
*Stephanonectriakeithii*	GJS92-133	-	AY489727	-	AY489622
*Sungiayongmunensis*	EFCC 2131	JN049856	EF468833	-	EF468770
*S.yongmunensis*	EFCC 2135	-	EF468834	-	EF468769
*Tilachlidiumbrachiatum*	CBS 506.67	KM231839	HQ232177	KM232415	KM231976
*T.brachiatum*	CBS 363.97	KM231838	KM231719	KM232414	KM231975
*Tolypocladiuminflatum*	SCALT1007-002	KC963032	-	-	-
*Trichodermaaggressivum*	CBS100525	-	JN939837	JQ014130	-
*T.arundinaceum*	ATCC 90237	EU330927	-	EU338326	EU338291
*T.viride*	GJS89-127	-	AY489726	-	AY489621
*Trichosphaerellaceratophora*	CBS 130.82	KM231847	KM231727	KM232423	KM231983
*Trichotheciumindicum*	CBS 123.78	-	NG_057651	-	-
*T.roseum*	DUCC 502	JN937590	JX458860	-	-
*Tyrannicordycepsfratricida*	TNS-F 19011	JQ349068	JQ257023	JQ257021	JQ257028
*Ustilaginoideadichromonae*	MRL IB9228	-	-	JQ257018	JQ257025
*U.virens*	ATCC 16180	-	-	JQ257019	JQ257026
*U.virens*	MAFF 240421	-	JQ257011	JQ257017	JQ257026
*Valetoniellopsislaxa*	GJS96-174	-	AY015635	AY015638	-
*Yosiokobayasiakusanagiensis*	TNS-F18494	-	JF415972	-	JF416014

Note: * J.F. White, Scale on Arundinaria tecta, North Carolina, 2000.

### ﻿Sequence alignment and phylogenetic analyses

Lasergene software (version 6.0, DNASTAR) was applied for the editing of DNA sequences in this study. The ITS, LSU, RPB2 and TEF sequences were downloaded from GenBank, based on Mongkolsamrit et al. (2018, 2020), Gao et al. (2021) and others selected on the basis of BLAST algorithm-based searches in GenBank (Table 1). A single gene data set was aligned and edited by MAFFT v7.037b (Katoh and Standley 2013) and MEGA v6.0 (Tamura et al. 2013). Combined sequences of ITS, LSU, RPB2 and TEF were performed by SequenceMatrix v.1.7.8 (Vaidya et al. 2011). The model was selected for Bayesian analysis by ModelFinder (Kalyaanamoorthy et al. 2017) in the software PhyloSuite v 1.2.2 (Zhang et al. 2020).

The combined genes were analyzed using Bayesian inference (BI) and maximum likelihood (ML) methods. For BI, a Markov chain Monte Carlo (MCMC) algorithm was used to generate phylogenetic trees with Bayesian probabilities using MrBayes v.3.2 (Ronquist et al. 2012) for the combined sequence datasets. The Bayesian analysis resulted in 20,001 trees after 10,000,000 generations. The first 4,000 trees, representing the burn-in phase of the analyses, were discarded, while the remaining 16,001 trees were used for calculating posterior probabilities in the majority rule consensus tree. After the analysis was finished, each run was examined using the program Tracer v1.5 (Drummond and Rambaut 2007) to determine burn-in and confirm that both runs had converged. ML analyses were constructed with IQ-TREE (Trifinopoulos et al. 2016) and the model was the default settings.

## ﻿Results

### ﻿Phylogenetic analyses

**Figure 1.**
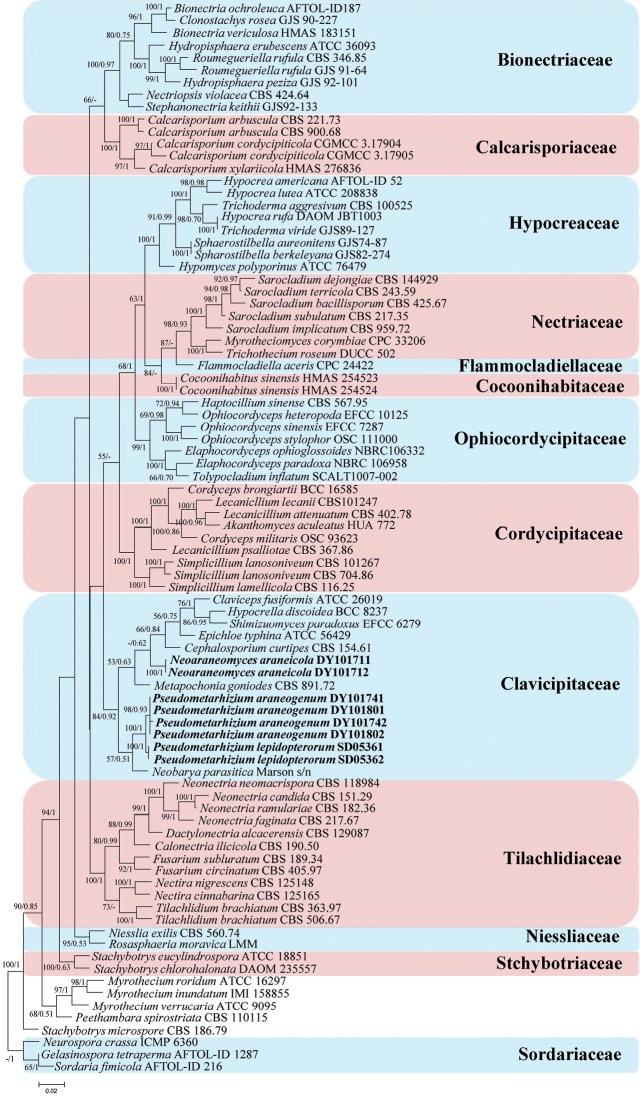
A maximum-likelihood phylogenetic tree of *Neoaraneomyces* and *Pseudometarhizium* in the order Hypocreales based on multigene dataset (ITS, LSU, RPB2 and TEF). Statistical support values (≥ 50%/0.5) are shown at the nodes for ML bootstrap support/BI posterior probabilities. The new taxa are in bold.

**Figure 2.**
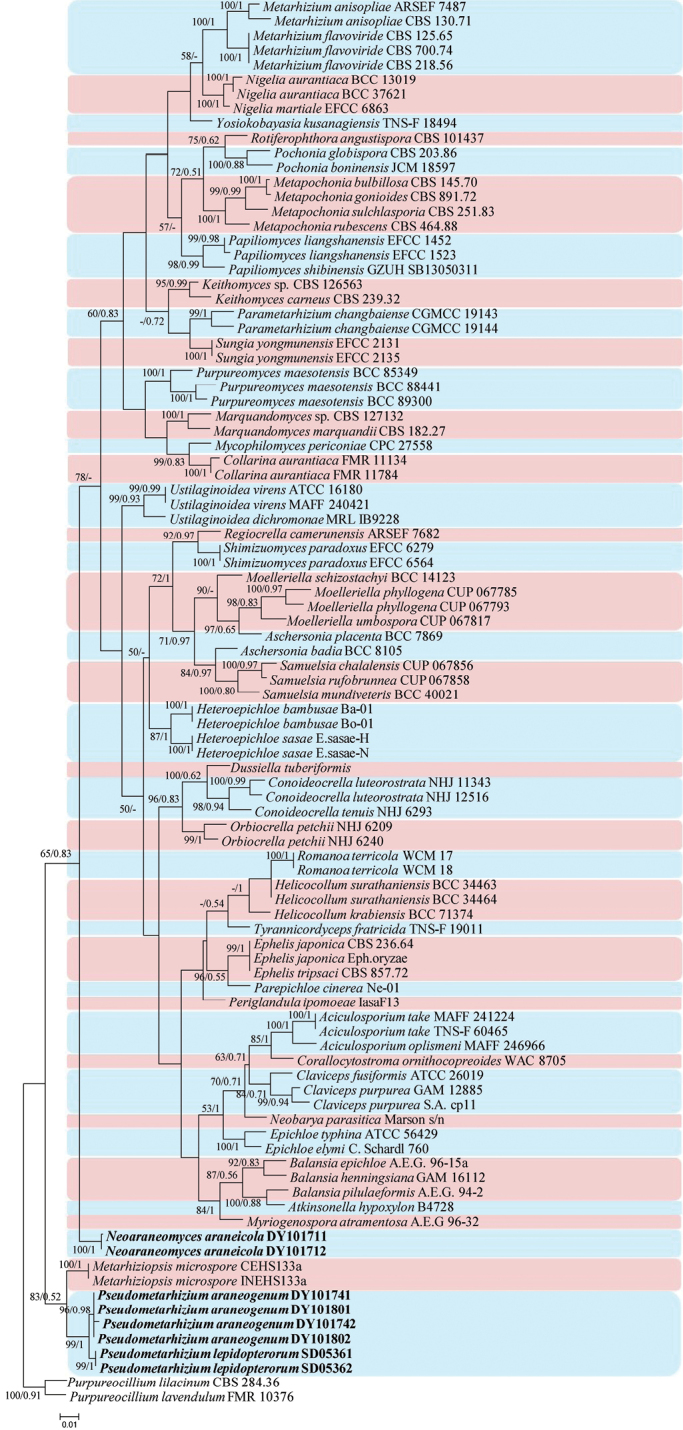
A maximum-likelihood phylogenetic tree of two new genera *Neoaraneomyces* and *Pseudometarhizium* and 39 genera in Clavicipitaceae, based on multigene dataset (ITS, LSU, RPB2 and TEF). Statistical support values (≥ 50%/0.5) are shown at the nodes for ML bootstrap support/BI posterior probabilities. The new taxa are in bold.

Phylogenetic trees were generated in analysis 1 (to determine the family placement of the new strains) and analysis 2 (to determine the establishment of the new genera in Clavicipitaceae) (Figs 1 and 2, respectively). *Gelasinosporatetrasperma* Dowding (AFTOL-ID 1287), *Neurosporacrassa* Shear & B.O. Dodge (ICMP 6360) and *Sordariafimicola* (Roberge ex Desm.) Ces. & De Not. (AFTOL-ID 216) were used as the outgroups in analysis 1, whereas *Purpureocilliumlilacinum* (Thom) Luangsa-ard, Houbraken, Hywel-Jones & Samson (CBS 284.36) and *P.lavendulum* Perdomo, Dania García, Gené, Cano & Guarro (FMR 10376) were used as the outgroups in analysis 2. The concatenated sequences of analysis 1 and 2 included 77 and 68 taxa, respectively, and consisted of 2,313 (ITS, 604; LSU, 570; RPB2, 576; and TEF, 563) and 2,470 (ITS, 583; LSU, 488; RPB2, 627; and TEF, 772) characters with gaps, respectively.

Analysis 1: The selected model for ML analysis was TIM2+F+I+G4. The final value of the highest scoring tree was –37,716.4419, which was obtained from an ML analysis of the dataset (ITS+LSU +RPB2+TEF). The parameters of the rate heterogeneity model used to analyze the dataset were estimated using the following frequencies: A = 0.2282, C = 0.2768, G = 0.2781, T = 0.2169; substitution rates AC = 1.4435, AG = 2.2494, AT = 1.4435, CG = 1.0000, CT = 5.4319 and GT = 1.0000, as well as the gamma distribution shape parameter α = 0.6711. The selected models for BI analysis were GTR+F+I+G4 (ITS, LSU and RPB2), and GTR+F+G4 (TEF). The phylogenetic trees (Fig. 1) constructed using ML and BI analyses were largely congruent and strongly supported in most branches. Each family was clustered into an independent clade. The new strains clustered into an independent clade (Clavicipitaceae) with close relationships to *Claviceps*, *Epichloe* (Fr.) Tul. & C. Tul., *Cephalosporium* Corda, *Metapochonia* Kepler, S.A. Rehner & Humber, *Hypocrella* Sacc. and *Shimizuomyces* Kobayasi.

Analysis 2: The final value of the highest scoring tree was –29,543.7455, which was obtained from the ML analysis of the dataset (ITS+LSU+RPB2+TEF). The parameters of the GTR model used to analyze the dataset were estimated based on the following frequencies: A = 0.2303, C = 0.2800, G = 0.2801, T = 0.2096; substitution rates AC = 1.0000, AG = 3.0029, AT = 1.0000, CG = 1.0000, CT = 7.0264 and GT = 1.0000, as well as the gamma distribution shape parameter α = 0.3934. The selected models for BI analysis were GTR+F+I+G4 (ITS+LSU+TEF) and SYM+G4 (RPB2). The phylogenetic trees (Fig. 2) constructed using ML and BI analyses were largely congruent and strongly supported in most branched. Most genera clustered into independent clades. Strains DY101711 and DY101712 clustered into an independent clade while DY101741, DY101742, DY101801, DY101802, SD05361 and SD05362 clustered into two independent clades with close relationship with *Metarhiziopsis* D.W. Li, R.S. Cowles & C.R. Vossbrinck.

### ﻿Taxonomy

#### ﻿ Neoaraneomyces

Taxon classificationFungiHypocrealesClavicipitaceae

W.H. Chen, Y.F. Han, J.D. Liang & Z.Q. Liang gen. nov.

9A86501A-8F6C-59C1-A432-474489DCAE86

 842644

##### Etymology.

Referring to a new genus parasitic on spiders

##### Type species.

*Neoaraneomycesaraneicola* W.H. Chen, Y.F. Han, J.D. Liang & Z.Q. Liang.

##### Description.

Colonies on PDA, white to grey, reverse yellowish. Conidiophores mononematous, usually arising from aerial hyphae, phialides solitary or in groups of two to three. Phialides emerging laterally from hyphae, forming a compact hymenium, abruptly narrowing into a neck. Conidia in chains, one-celled, hyaline, fusiform or ellipsoidal.

##### Host.

Spider (Araneidae)

##### Habitat.

Near roads and located on or under rocks.

##### Sexual morph.

Unknown.

##### Notes.

The genera *Akanthomyces*, *Beauveria*, *Clonostachys*, *Cordyceps*, *Engyodontium* de Hoog, *Gibellula*, *Hevansia*, *Hirsutella*, *Hymenostilbe*, *Lecanicillium* W. Gams & Zare, *Ophiocordyceps* Petch, *Purpureocillium*, and *Torrubiella* Boud. have been reported as spider-pathogenic fungi in Hypocreales (Shrestha et al. 2019). *Gibellula* is only found on spiders. *Neoaraneomyces* differs from *Gibellua* by its paecilomyces-like conidiogenous structures, phialides which were solitary or in groups of two to four, with fusiform to ellipsoidal conidia.

#### ﻿ Neoaraneomyces araneicola

Taxon classificationFungiHypocrealesClavicipitaceae

W.H. Chen, Y.F. Han, J.D. Liang & Z.Q. Liang sp. nov.

5EA97D06-6C0C-5FDB-8D4A-44194D2C0B46

 842645

Fig. 3

##### Type.

Duyun City (26°21'27.96"N, 107°22'48.22"E), Qiannan Buyi and Miao Autonomous Prefecture, Guizhou, China. On a dead spider (Araneae), 1 October 2019, Wanhao Chen, GZAC DY10171 (holotype); ex-type living cultures, DY101711.

**Figure 3.**
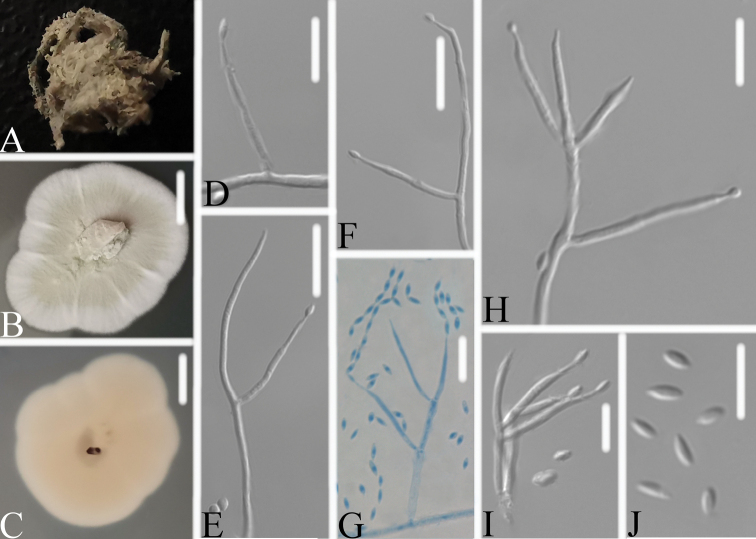
*Neoaraneomycesaraneicola***A** infected spider **B, C**PDA-containing culture plate showing **B** the front and **C** reverse sides of the colony **D–J** phialides, conidia in chains and conidia. Scale bars: 10 mm (**B, C**); 10 μm (**D–J**).

##### Description.

Spider host completely covered by white mycelium. Conidiophores mononematous, arising from the lateral hyphae. Colonies on PDA, 3.0–3.2 cm diam. after 14 d at 25 °C, white to pale grey, powdery, consisting of a basal felt, reverse yellowish. Prostrate hyphae smooth, septate, hyaline, 1.4–2.2 μm diam. Erect conidiophores usually arising from aerial hyphae. Phialides single or in groups of two to three, 8.9–23.8 × 1.1–1.6 μm, with a cylindrical to ellipsoidal basal portion, tapering into a short distinct neck. Conidia in chains, hyaline, fusiform to ellipsoidal, one-celled, 2.9–4.4 × 1.3–2.0 μm. Sexual state not observed.

##### Host.

Spider (Araneidae).

##### Habitat.

Near the road, located on or under rocks.

##### Etymology.

Referring to the ability to colonize spiders.

##### Additional strain examined.

Duyun City (26°21'27.96"N, 107°22'48.22"E), Qiannan Buyi and Miao Autonomous Prefecture, Guizhou, China. On a dead spider (Araneae), 1 October 2019, Wanhao Chen, DY101712.

#### ﻿ Pseudometarhizium

Taxon classificationFungiHypocrealesClavicipitaceae

W.H. Chen, Y.F. Han, J.D. Liang & Z.Q. Liang gen. nov.

B03C529B-D645-587A-ACF4-5A5612BB2C01

 842641

##### Etymology.

Referring to *Metarhizium*-like colony.

##### Type species.

*Pseudometarhiziumaraneogenum* W.H. Chen, Y.F. Han, J.D. Liang & Z.Q. Liang.

##### Description.

Colonies on PDA, light green, reserve brown to light brown. Conidiophores synnematous or mononematous, erect, scattered. Phialides emerging laterally from synnemata or hyphae, forming a compact hymenium, abruptly narrowing into a helical neck. Conidia, one-celled, fusiform or ellipsoidal.

##### Host.

Spider (Araneae).

##### Habitat.

Near the road, located on or under rocks, or on the underside of leaves of broad-leaved plant species.

##### Sexual morph.

Unknown.

##### Notes.

The light green colonies of *Pseudometarhizium* are similar to those of *Metarhizium* species. However, *Pseudometarhizium* is easily distinguished by the combined datasets (ITS+LSU+RPB2+TEF), and had a close relationship with *Metarhiziopsis*. *Pseudometarhizium* can be easily distinguished from *Metarhiziopsis* by its paecilomyces-like structure and absence of sporodochia.

#### ﻿ Pseudometarhizium araneogenum

Taxon classificationFungiHypocrealesClavicipitaceae

W.H. Chen, Y.F. Han, J.D. Liang & Z.Q. Liang sp. nov.

3C9E4460-D658-533A-8CA7-1BB2D91AED2D

 842642

Fig. 4

##### Type.

Duyun City (26°21'27.96"N, 107°22'48.22"E), Qiannan Buyi and Miao Autonomous Prefecture, Guizhou, China. On a dead spider (Araneae), 1 October 2019, Wanhao Chen, GZAC DY10180 (holotype), ex-type living cultures, DY101801.

**Figure 4.**
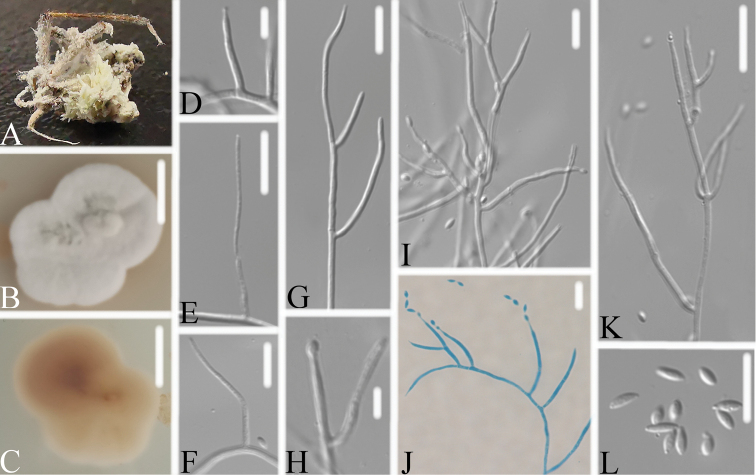
*Pseudometarhiziumaraneogenum***A** infected spider **B, C** culture growing on PDA, **B** front and **C** the reverse sides of the colony **D–L** solitary phialides, or groups of two, conidia in short chains and individual. Scale bars: 10 mm (**B, C**); 10 μm (**D–L**).

##### Description.

Spider host completely covered by white mycelium. Conidiophores mononematous, arise from the lateral hyphae. Colonies irregularly on PDA, 1.8–2.8 cm diam. after 14 d at 25 °C, white, consisting of a basal felt, floccose hyphal overgrowth, reverse yellowish to pale brown or green. Prostrate hyphae smooth, septate, hyaline, 1.0–1.2 μm diam. Erect conidiophores usually arising from aerial hyphae. Phialides solitary or in groups of two, 8.3–23.3 × 1.3–2.2 μm, with a cylindrical basal portion, tapering into a short distinct neck. Conidia in chains, hyaline, fusiform, one-celled, 3.4–5.8 × 1.4–1.8 μm. Sexual state not observed.

##### Host.

Spider (Araneidae).

##### Habitat.

Near the road, located on or under rocks.

##### Etymology.

Referring to the ability to colonize spiders.

##### Additional specimen examined.

Duyun City (26°21'27.96"N, 107°22'48.22"E) Qiannan Buyi and Miao Autonomous Prefecture, Guizhou, China. On a dead spider (Araneae), 1 October 2019, Wanhao Chen, GZAC DY10174, living cultures, DY101741, DY101742.

##### Remarks.

*Pseudometarhiziumaraneogenum* distinguished from *P.lepidopterorum*, which has longer phialides (21.2–33.7 × 1.1–1.4 μm) and smaller conidia (3.1–4.3 × 1.3–1.5 μm).

#### ﻿ Pseudometarhizium lepidopterorum

Taxon classificationFungiHypocrealesClavicipitaceae

W.H. Chen, Y.F. Han, J.D. Liang & Z.Q. Liang sp. nov.

2B50F334-4B0D-584D-BFB0-0F52CC0FB617

 842643

Fig. 5

##### Type.

Sandu County (25°57'22.21"N, 107°57'54.69"E), Qiannan Buyi and Miao Autonomous Prefecture, Guizhou, China. On a pupa (Lepidoptera), 1 May 2019, Wanhao Chen, GZAC SD0536 (holotype), ex-type living cultures, SD05361.

**Figure 5.**
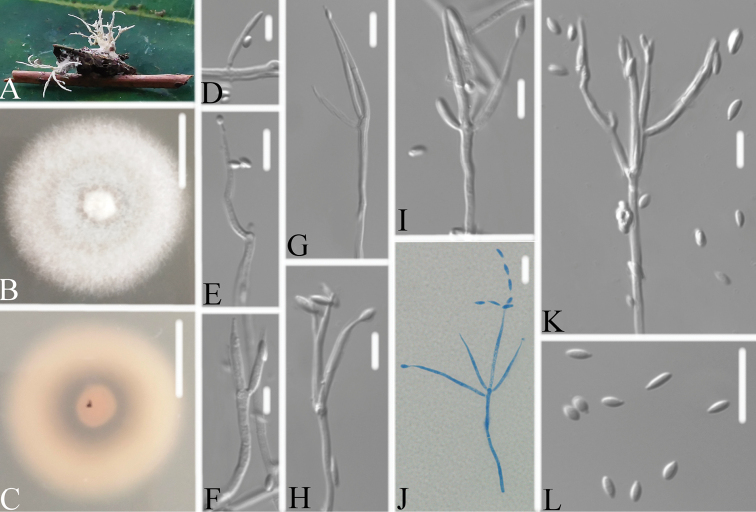
*Pseudometarhiziumlepidopterorum***A** infected pupa (Lepidoptera) **B, C** culture on PDA showing **B** front and **C** reverse sides of the colony **D–L** solitary phialides, or groups of two to three, and conidia in short chains and individual. Scale bars: 10 mm (**B, C**); 10 μm (**D–L**).

**Description.** Host pupa completely covered by white mycelium. Conidiophores arising from lateral hyphae of the synnemata. Colonies on PDA, 1.4–2.0 cm diam. after 14 d at 25 °C, white, consisting of a basal felt and cottony, floccose hyphal overgrowth, reverse yellowish to pale green. Prostrate hyphae smooth, septate, hyaline, 1.0–2.0 μm diam. Erect conidiophores usually arising from aerial hyphae. Phialides solitary or in groups of two to three, 21.2–33.7 × 1.1–1.4 μm, with a cylindrical basal portion, tapering into a short distinct neck. Conidia in chains, hyaline, fusiform, one-celled, 3.1–4.3 × 1.3–1.5 μm. Sexual state not observed.

##### Host.

Pupa (Lepidoptera).

##### Habitat.

On the underside of leaves of broad-leaved plant species.

##### Additional strain examined.

Sandu County (25°57'22.21"N, 107°57'54.69"E) Qiannan Buyi and Miao Autonomous Prefecture, Guizhou, China. On a pupa (Lepidoptera), 1 May 2019, Wanhao Chen, SD05362.

##### Etymology.

Referring to its insect host, order Lepidoptera.

##### Remarks.

*Pseudometarhiziumlepidopterorum* distinguished from *P.araneogenum*, which has shorter phialides (8.3–23.3 × 1.3–2.2 μm) and longer conidia (3.4–5.8 × 1.4–1.8 μm).

## ﻿Discussion

Paecilomyces-like conidiogenous structure is common throughout the Hypocreales (Luangsa-ard et al. 2004) and their presence in the new strains make it impossible to identify them using only morphological characteristics. To determine the family placement of the new strains, a phylogenetic tree was constructed with the combined dataset (ITS+LSU+RPB2+TEF) for 14 families of Hypocreales. The new strains clustered into the Clavicipitaceae clade, confirming that they belonged to this family.

Currently, Clavicipitaceae contains 49 genera (Hyde et al. 2020; Mongkolsamrit et al. 2020; Gao et al. 2021). A phylogenetic analysis was carried out based on the available sequences from 39 of these genera. The new strains clustered into independent clades, suggesting that they belong to new genera in the family Clavicipitaceae. Among the genera without available sequences, *Helminthascus* Tranzschel and *Sphaerocordyceps* Kobayasi are spider- and insect-associated teleomorph genera without an asexual state (Hyde et al. 2020). The new strains were easily distinguished from *Helminthascus* and *Sphaerocordyceps* by their absence of a teleomorph state and pale green color in the natural state. Thus, the new strains are described as two new genera, based on phylogenetic analysis and morphological characteristics.

The evolutionary dynamics of fungi and their hosts are usually described either through coevolution or host shifts (Vega et al. 2009). In a common ecological niche, shifts to new hosts often occur in accordance with the fungal nutrient requirements. The common ancestor of Hypocreaceae and Clavicipitaceae corresponds to a departure from plant-based nutrition to a model that specializes in animals and fungi (Spatafora et al. 2007). Clavicipitaceous fungi, especially those of the genus *Metarhizium*, are pathogenic to scale insects, white flies and other insect orders. However, few spider-associated species have been reported. Based on comparison of their evolutional relationships with close relatives, we hypothesize that the new spider-associated genera might have undergone host jumps or transferred their nutritional preferences.

Both mononematous and synnematous conidiophores were reported in natural conditions in the present study. Synnematous entomopathogenic fungi (such as *Gibellula* spp.) are found on the abaxial leaf surfaces of shrubbery, forest floors and shallow soil layers (Hywel-Jones 1996). These entomopathogenic fungi do not spread by airflow diffusion but employ particular strategies, such as producing synnemata and sticky conidia, to accommodate various arthropod activities and facilitate conidial spread (Abbott 2002). In contrast, strains with mononematous conidiophores occur in more open portions of forests and favor dry conidial dispersal (Chen et al. 2020). *Pseudometarhiziumlepidopterorum* was found on the undersides of leaves of broad-leaved plant species, whereas *Neoaraneomycesaraneicola* and *P.araneogenum* were found near the road and were located on or under rocks. Thus, we speculate that the presence of synnemata may be the result of convergent evolution to adapt to the ecological environment.

## Supplementary Material

XML Treatment for Neoaraneomyces

XML Treatment for Neoaraneomyces araneicola

XML Treatment for Pseudometarhizium

XML Treatment for Pseudometarhizium araneogenum

XML Treatment for Pseudometarhizium lepidopterorum

## Supplementary materials

Supplementary material 1 Table S1 Data type COL. Explanation note Primers information for 5 DNA sequences. https://binary.pensoft.net/file/711835This dataset is made available under the Open Database License (http://opendatacommons.org/licenses/odbl/1.0/). The Open Database License (ODbL) is a license agreement intended to allow users to freely share, modify, and use this Dataset while maintaining this same freedom for others, provided that the original source and author(s) are credited.Wan-Hao Chen, Jian-Dong Liang, Xiu-Xiu Ren, Jie-Hong Zhao, Yan-Feng Han, Zong-Qi Liang
