# Supplementary material for: ﻿Phylogenetic, ecological and morphological characteristics reveal two new spider-associated genera in Clavicipitaceae
Source: MycoKeys. 2022 Jul 7;91:49–66. doi: 10.3897/mycokeys.91.86812 (PMC9849053; doi:10.3897/mycokeys.91.86812)
Supplement: Supplementary material 1 — Table S1 [file mycokeys-91-049-s001.pdf]

Table S1. Primers information for 5 DNA sequences

Name	Length	Direction	Sequence 5´-3´
ITS5	22	forward	GGAAGTAAAAGTCGTAACAAGG
ITS4	20	reverse	TCCTCCGCTTATTGATATGC
LROR	17	forward	ACCCGCTGAACTTAAGC
LR5	17	reverse	TCCTGAGGGAAACTTCG
CRPB1	20	forward	CAYCCWGGYTTYATCAAGAA
RPB1Cr	23	reverse	CCNGCDATNTCRTTRTCCATRTA
RPB2-5F3	20	forward	GACGACCGTGATCACTTTGG
RPB2-7Cr2	20	reverse	CCCATGGCCTGTTTGCCCAT
983F	23	forward	GCYCCYGGHCAYCGTGAYTTYAT
2218R	23	reverse	ATGACACCRACRGCRACRGTYTG
